# Use and abuse of fecal occult blood tests: a community hospital experience

**DOI:** 10.1186/s12876-019-1079-9

**Published:** 2019-09-03

**Authors:** Sarthak Soin, Olalekan Akanbi, Abdullah Ahmed, Yunha Kim, Sarbagya Pandit, Igor Wroblewski, Nasir Saleem

**Affiliations:** 1 Department of Internal Medicine, Amitahealth Saint Joseph Hospital in Affiliation with University of Illinois College of Medicine, Chicago, Il 60657 USA; 2 Division of Hospital Medicine, University of Kentucky, Lexington, KY 40536 USA; 3 Department of Gastroenterology and Hepatology, University of Tennessee Health Science Center, Memphis, TN USA

**Keywords:** Colorectal cancer, Gastrointestinal bleeding, Screening, Fecal occult blood test

## Abstract

**Background:**

The Fecal Occult Blood Test (FOBT) is one of the diagnostic modalities indicated for screening patients for Colorectal Cancer (CRC). Despite being approved only for screening for CRC, numerous studies in the past have illustrated misuse of the FOBT. We examined utilization of the FOBT for patients admitted to a community teaching hospital.

**Methods:**

The study was conducted at Saint Joseph Hospital, Chicago USA. A retrospective review of Electronic Medical Records (EMRs) of patients admitted from January 2016 to December 2017 was performed.

**Results:**

We reviewed the EMRs of 729 patients who received the stool testing for occult blood (FOBT). All tests (100%) were carried out for purposes other than CRC screening. Anemia (38%) was the most common reason documented for carrying out the FOBT. Further, 88% of the tests were ordered on patients who either did not fulfill CRC screening criteria or had other contraindications for testing. Usage of contraindicated medication was the most important factor (58% of patients) that made the candidates ineligible for testing. A total 73 Colonoscopies were ordered for patients who received the test inappropriately with a resulting low yield (0.47%) of CRC diagnosis.

**Conclusion:**

The stool occult blood test continues to be utilized for reasons other than CRC screening. Majority of patients who underwent the test were not suitable candidates due to the presence of contraindications for testing. Unsuitable FOBT testing led to further unnecessary investigations.

## Background

Colorectal cancer (CRC) is the third most common cancer worldwide and one of the leading causes of cancer-related mortality [1]. In the United States of America (USA), it is the third most common cause of cancer related deaths [2]. With such a high burden, CRC has deservedly received attention not just from healthcare providers but also from policy makers to devise effective strategies to increase screening, diagnosis and reduce mortality [3]. Screening (primary prevention) is an essential component in the fight against CRC. Various investigations are available for screening patients; Colonoscopy, flexible Sigmoidoscopy, Fecal Occult Blood Test (FOBT) and CT Colonography.

According to the guidelines issued by American Cancer Society (ACS), FOBT has been approved for screening patients (age 45 or above) who are at average risk of being diagnosed with CRC [4]. FOBT involves collecting and analyzing a stool sample for the presence of non-apparent blood, which might indicate an underlying CRC. The test is inexpensive, non-invasive and has in some cases demonstrates good patient compliance. There are multiple types of the FOBT; the guaiac-based FOBT (gFOBT) is designed to detect the activity of the enzyme known as Peroxidase (found in human red blood cells); the immunochemical based FOBT (FIT), relies on detection of the protein called Globin (found in human red blood cells). The concept of testing stool samples for presence of occult blood started in the early 1900s and numerous studies since then have documented the efficacy of the FOBT (in conjunction with a follow up Colonoscopy in case of a positive result) in uncovering the presence of CRC [5–12].

Moreover, randomized control trials have showed a decrease in CRC-related mortality with the use of FOBT [13–16]. With its documented efficacy as a screening tool, ability to lower CRC related mortality, low cost and non-invasive nature, the FOBT has gained widespread use and unfortunately, a potential for abuse. Multiple prior studies, mostly in large academic centers, have shown inappropriate use of FOBT in patients not meeting criteria for CRC screening and as an adjunctive diagnostic modality in other clinical scenarios, most notably in workup for possible gastrointestinal bleeding [17–20]. Hence, with this study, we sought to assess the utilization of FOBT in a community hospital setting.

## Methods

We chose a retrospective observational investigation model for our study. It involved conducting a chart review for patients admitted to Saint Joseph Hospital from January the 1st 2016 till December the 31st 2017. Electronic medical charts were reviewed for all patients admitted to the emergency room, general medical floor, telemetry and the intensive care unit who received the gFOBT (Hemoccult II, Beckman Coulter, CA, USA).

Three internal medicine resident physician investigators were assigned the task of conducting a review of medical charts (from an electronic health records system) for patients who received FOBT. After selection of eligible charts, a detailed chart review was performed, and data was collected. The three investigators were not involved in delivering clinical care for the patients they were assigned to review charts for our study.

A standard questionnaire was used to collect predetermined data points. The questionnaire was designed to extract information in the following categories: patient demographics and historical data; the performance of a digital rectal exam (DRE) and/or a FOBT while being admitted to the hospital, along with their results; follow up procedures for the patients with positive FOBT results and their subsequent outcomes. The detail of the data that was supposed to be collected from every patient’s chart is presented in Table 1.

**Table 1:** Standardized questionnaire used for collection of data

Patient demographics and historical data.	
Age	
Gender	
Presenting complain	
Presence of signs of Gastrointestinal bleeding on admission to the hospital such as; Hematemesis, Coffee-ground emesis, Melena, Hematochezia, bright blood per rectum.	
Past history of Gastrointestinal bleeding	
Past history of; Colorectal polyps, Colorectal cancer	
Past history of Gastrointestinal related procedures; Barium enema, Colonoscopy etc.	
Family history of Colorectal cancer	
Recent usage of medications that are contraindicated in patients that undergo the FOBT (aspirin, antiplatelets, non-steroidal anti-inflammatory drugs, anticoagulants, Vitamin C)	
Performance of a digital rectal exam (DRE) and/or FOBT while being admitted to the Hospital, along with their results.	
Performance and findings of DRE	
Use of DRE to obtain stool sample for FOBT	
Performance and results of FOBT (gFOBT)	
Rationale for using FOBT	
Repetition of DRE and FOBT	
Follow up procedures for the patients with positive FOBT results and their subsequent outcomes.	
Gastroenterology service consultation	
Follow up investigations undertaken	
Results of follow up investigations	

Appropriateness of FOBT was assessed by utilizing the prespecified criteria: age from 50 to 75 years, absence of active bleeding from the gastrointestinal tract on admission or during hospitalization, no recent usage of contraindicated medication (aspirin, antiplatelets, non-steroidal anti-inflammatory drugs, anticoagulants, Vitamin C) and no previous history of gastrointestinal bleeding, colonic polyps or CRC. In our study we define potential appropriateness as population that would have been eligible for colorectal cancer screening as per current recommended guidelines.

The data collected from the protocol was saved on a Microsoft excel spreadsheet and subsequent descriptive analysis was performed using SPSS 25.0 by IBM.

## Results

We identified 729 patients who received FOBT among which 290 patients (39.8%) had positive results. There were 317 males (43.5%) and 412 females (56.5%) with a mean age of 65 years (range 21–105). Most of these identified patients were on the general medical floor (Table 2). The most common stated indication for FOBT was evidence of anemia on laboratory findings (38.4%). Other reasons for ordering the FOBT included abdominal pain, bright red blood per rectum, melena, coffee ground emesis, hematemesis, shortness of breath, syncope, sepsis, nausea, vomiting and fatigue (Table 3).

**Table 2:** Patient Demographics and place of performance of the FOBT in the Hospital

Age (mean): 64.4 years.	
Gender: Males: 317 patients (43.5%), Females: 412 patients (56.5%).	
FOBT performed in the ER: 130 patients (17.8%)	
FOBT performed on the General Medical Floor: 268 patients (36.8%)	
FOBT performed on the Telemetry Floor: 223 (30.6%)	
FOBT performed in the Intensive Care Unit: 108 (14.8%)

**Table 3:** Indications for fecal occult blood testing

• Anemia	280 (38.4%)
• Melena	81 (11.1%)
• Abdominal pain	63 (8.6%)
• Lower GI bleeding	112 (15.3%)
• Coffee ground emesis	17 (2.3%)
• Others (Shortness of breath, syncope, sepsis, nausea, vomiting and fatigue)	176 (24.3%)

**Fig. 1:**
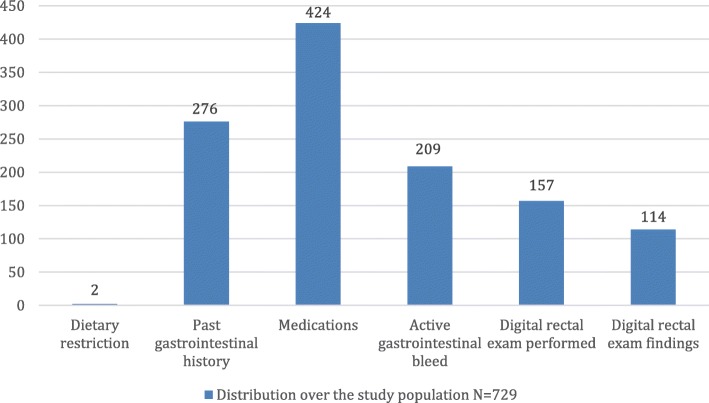
Composite analysis showing distribution of various findings among the entire study population

**Fig. 2:**
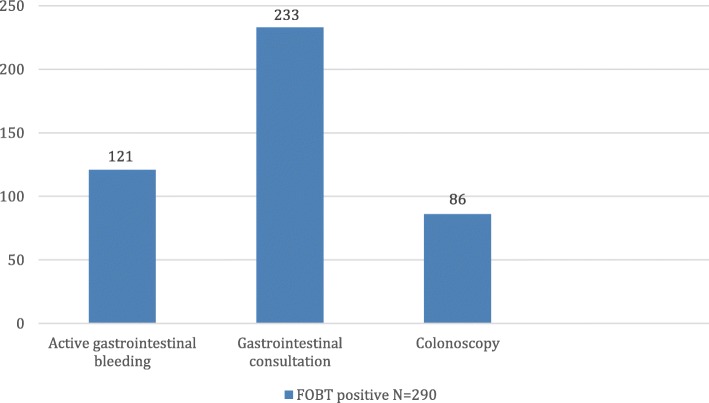
Sub analysis of the FOBT positive population showing distribution of gastroenterology consultation, active GI bleeding and colonoscopies

Furthermore, 424 patients (58.2%) had recent usage of contraindicated medicines; 209 patients (28.7%) had evidence of active bleeding from the gastrointestinal tract; 276 patients (37.9%) had a significant past history of gastrointestinal bleeding, history of colonic polyps or history of colorectal cancer. 105 patients (14.4%) had their stool sample collected during DRE. Only 2 cases of dietary restrictions were evident from the review of records (Fig. 1). Notably, 121 patients (41.7%) of those who tested positive with the FOBT had active gastrointestinal bleeding while 233 patients (80.3%) were referred to the gastrointestinal consult service (Fig. 2).

**Fig. 3:**
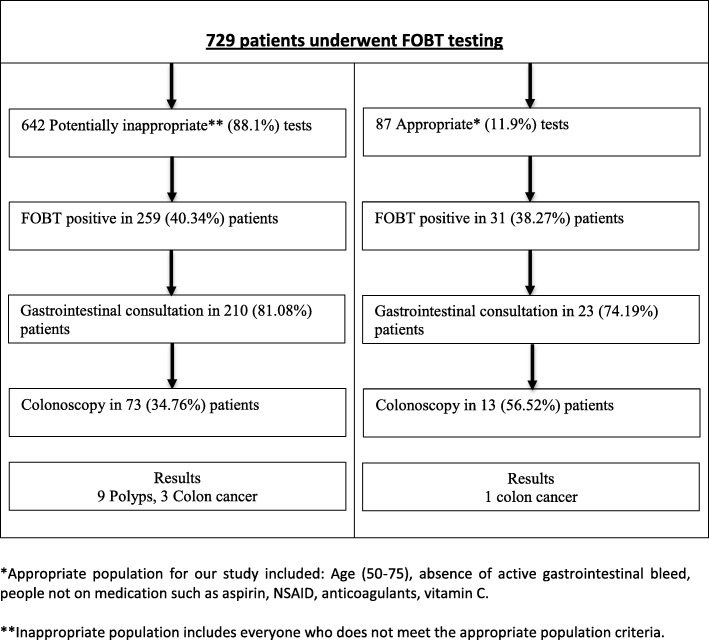
Final diagnostic yield among the appropriate and inappropriate population. *Appropriate population for our study included: Age (50–75), absence of active gastrointestinal bleed, people not on medication such as aspirin, NSAID, anticoagulants, vitamin C. **Inappropriate population includes everyone who does not meet the appropriate population criteria

FOBT testing was applied inappropriately to 642 (out of 729) patients (88.1%), this was labeled as the ‘potentially inappropriate’ group. Thus only 87 patients (11.9%) who received the test fulfilled our prespecified criteria of appropriateness and were labeled as the ‘potentially appropriate’ group. Of the 642 potentially inappropriate cohort, 259 had a positive FOBT among which 73 underwent a diagnostic colonoscopy, yielding only 3 cases of CRC (0.47%) (Table 4). For remaining 186 patient’s colonoscopies were not pursued after discussion with the gastroenterology service and/or if there was high likelihood of false positive FOBT. Hence there was a ratio of 1 case of CRC diagnosed for every 214 patients inappropriately tested with the FOBT. There were 31 positive FOBT results from the cohort of 87 patients who were potentially appropriately tested. Thirteen proceeded to a colonoscopy with a diagnostic yield of 1 colon cancer (1.14%) (Fig. 3).

**Table 4:** Breakdown of the study population based on the appropriateness of FOBT testing

Potentially appropriate population 87 (11.9%)	Potentially inappropriate population 642 (88.1%)
• 87 patients aged 50–75	• 125 patients aged < 50
• Not taking medications known to alter testing (NSAIDS, anticoagulants, aspirin, vitamin C)	• 225 patients aged > 75
• No patients with active gastrointestinal bleed	• 247 patients on medication between age group 50–75 (NSAIDS, aspirin, anticoagulants and vitamin C)
• FOBT positive in 31/87 patients (38.27%)	• 45 patients between 50 and 75 with active gastrointestinal bleeding
• 23/31 (74.19%) patients had gastrointestinal consult	• FOBT positive 259/642 (40.34%) patients
• 13/31 (41.93%) patients had colonoscopy	• 210/259 (81.08%) patients had gastrointestinal consultation
	• 73/259 (28.18%) patients underwent colonoscopy

## Discussion

Using primary data obtained in a community hospital setting, we performed a retrospective analysis evaluating the use and misuse of fecal occult blood tests among inpatients. Our study revealed a large burden of potentially inappropriate utilization of FOBT with abysmally low diagnostic yield. During the study period, we collected data on 729 patients who received the FOBT. The sample size of our study is more as compared to some previous studies [21]. Hence the results of our study might be more representative of the actual practices regarding the FOBT.

One of the starkest findings from our study was that not a single FOBT was ordered for the documented reason for screening CRC in the hospital. Instead, anemia was the most common reason documented for ordering the FOBT in the charts; findings that echo the results of other studies [21, 22]. Another important reason documented in the charts for ordering the FOBT was to check for gastrointestinal bleeding. Despite the fact that FOBT has been only approved for screening patients for CRC, it was largely used for various other purposes in hospitalized patients.

As illustrated above, many studies previously had documented the incorrect application of the FOBT [22–24] and the results of our study are consistent with them. Not only did our results showed that FOBTs ordered were not intended for CRC screening, we found that 88.1% of patients who received the test were not suitable candidates based on the presence of specific contraindications. This is a huge percentage for the potentially inappropriate group and represents a large magnitude of misuse. The proportion of patients who received the test in unsuitable conditions in previous studies (such as the ones done) by Powel et al. and Sharma et al. [24, 25] was not as high as the 88.1% that we obtained from our results. The most common factor that made patients non-candidates was the use of contraindicated medications prior to the FOBT, which was the case in around 58% of patients tested in our study. This result is in line with other studies done by Friedman et al. and Narula et al. [18, 21] that also showed that a significantly large proportion of the patients were already on non-recommended medications before they undertook the FOBT.

The DRE is an essential component of a complete physical examination. It should be carried out on every patient that is admitted to the hospital with gastrointestinal-related illness unless contraindicated. One of the advantages of carrying out a DRE is that it can show signs of acute gastrointestinal bleeding (based only on the appearance of the stool sample). Our results show that only 157 patients (from 729 patient charts reviewed) got a DRE on admission. This might be one of the reasons for the high rates of inappropriate ordering of the FOBT as many clinicians might have wanted to find signs of gastrointestinal bleeding through the FOBT results and not from the stool appearance of the DRE. The study from Sharma et al. [25] also showed similar decreasing trends of doing a DRE for patients admitted to the hospital.

The practice of ordering the FOBT inappropriately was found to be prevalent in all departments (Emergency Department, General Medical Floor and Intensive Care Units) included in our study. The study conducted by Nadel et al. [20] had shown that primary care physicians are also involved in ordering the FOBT for their patients under unsuitable conditions. Thus, combining the results of our study with that of Nadel et al., indicates that physicians from multiple specialties; Family Medicine, Internal Medicine, Emergency Medicine and Critical Care Medicine are complicit of not following the guidelines when ordering the test.

This study also highlights the burden of unnecessary procedures as a result of inappropriate FOBT testing. A total of 73 colonoscopies were done in patients who tested positive for the FOBT from the potentially inappropriate group. A total of 3 colon cancer were found in the inappropriate group, although it is important to consider none of the FOBT testing was performed for this reason specifically. The study done by Mosadeghi et al. [19] showed that the odds of getting further investigations are much higher when the results of the FOBT done for any reason is positive. Taking this finding and using it with our results, it is highly probable that if inappropriate FOBTs were not done for our patients, we would have had a smaller number of positive FOBT results leading to a fewer amounts of performed colonoscopies potentially leading to lower hospitalization cost and length of stay. Resources, including manpower and healthcare dollars are limited and should be best utilized where there is a genuine need.

Besides the point that none of the patients in our study was tested by the FOBT with the intention of CRC screening, we thought it would still be interesting to note the occurrence of CRC in the potentially inappropriate and potentially appropriate groups. We calculated that for every 214 inappropriately tested patients, 1 case of CRC was diagnosed. If we see the data for the potentially appropriate patient tested, we see that ratio is expectantly lesser; 1 case of CRC diagnosed for every 87 patients. The fact that very few cases of CRC were detected in the potentially inappropriate groups is more proof that further investigations in the inappropriately tested group did not add to any significant clinical benefit.

Like all clinical investigations, our study had some inherent strengths and limitations. Our large sample size and data collection spanning 2 years for the entire hospital (not just one department), gives us confidence that the results of our study could represent the actual practices regarding the use of the FOBT at a community hospital setting. Having said that, this was a retrospective observational investigation; hence the results, analysis and conclusions of our study should be interpreted in the context of limitations associated with such studies. Also, of note is that at the time of data collection, data entry and data analysis (January 2018); the criteria we used to check the eligibility of patients for the FOBT was age above 50 years. The American Cancer Society (ACS) revised the age criteria for CRC screening to be above the age of 45 (it used to be above 50 years previously) in May 2018. Hence some patients from our results (those between the ages 45–50) might have been mislabeled as potentially inappropriate for the FOBT screening based on the latest criteria issued by the ACS. However, this is unlikely to affect the results and trends that our study showed regarding the inappropriate use of the FOBT significantly.

## Conclusion

This study further emphasizes the potential for misuse of the FOBT for purposes other than CRC screening in hospitalized patients who are mostly unsuitable for this test. Excessive use of the FOBT results in further unnecessary investigations with abysmally low diagnostic yields. Judicious use of this test as recommended may minimize healthcare utilization of inpatients.

## Data Availability

The datasets used and/or analyzed during the current study available from the corresponding author on request.

## Abbreviations

ACS: American Cancer Society
CRC: Colorectal Cancer
EMRs: Electronic Medical Records
FOBT: Fecal Occult Blood Test
USA: United States of America
